# A sub-national real-time epidemiological and vaccination database for the COVID-19 pandemic in Canada

**DOI:** 10.1038/s41597-021-00955-2

**Published:** 2021-07-15

**Authors:** Isha Berry, Meghan O’Neill, Shelby L. Sturrock, James E. Wright, Kamal Acharya, Gabrielle Brankston, Vinyas Harish, Kathy Kornas, Nika Maani, Thivya Naganathan, Lindsay Obress, Tanya Rossi, Alison E. Simmons, Matthew Van Camp, Xiao Xie, Ashleigh R. Tuite, Amy L. Greer, David N. Fisman, Jean-Paul R. Soucy

**Affiliations:** 1 Dalla Lana School of Public Health, University of Toronto, Toronto, Ontario Canada; 2 Centre for Global Child Health, The Hospital for Sick Children, Toronto, Ontario Canada; 3 Department of Population Medicine, Ontario Veterinary College, University of Guelph, Guelph, Ontario Canada; 4 Faculty of Medicine, University of Toronto, Toronto, Ontario Canada; 5 Department of Molecular Genetics, University of Toronto, Toronto, Ontario Canada

**Keywords:** Geography, Infectious diseases

## Abstract

The COVID-19 pandemic has demonstrated the need for real-time, open-access epidemiological information to inform public health decision-making and outbreak control efforts. In Canada, authority for healthcare delivery primarily lies at the provincial and territorial level; however, at the outset of the pandemic no definitive pan-Canadian COVID-19 datasets were available. The COVID-19 Canada Open Data Working Group was created to fill this crucial data gap. As a team of volunteer contributors, we collect daily COVID-19 data from a variety of governmental and non-governmental sources and curate a line-list of cases and mortality for all provinces and territories of Canada, including information on location, age, sex, travel history, and exposure, where available. We also curate time series of COVID-19 recoveries, testing, and vaccine doses administered and distributed. Data are recorded systematically at a fine sub-national scale, which can be used to support robust understanding of COVID-19 hotspots. We continue to maintain this dataset, and an accompanying online dashboard, to provide a reliable pan-Canadian COVID-19 resource to researchers, journalists, and the general public.

## Abstract (summary)

**Table:** 

Measurement(s)	COVID-19 Cases • COVID-19 Mortalities • COVID-19 vaccination
Technology Type(s)	digital curation
Sample Characteristic - Organism	Homo sapiens
Sample Characteristic - Location	Canada

Machine-accessible metadata file describing the reported data: 10.6084/m9.figshare.14331311

## Background & Summary

Coronavirus Disease 2019 (COVID-19) was first detected in December 2019 in Wuhan, China and has since developed into a pandemic causing substantial global morbidity and mortality^1^. This pandemic has emphasized the need for rapid, accurate, and open-access data to support real-time evidence-based decision making and outbreak control efforts^2,3^. In particular, individual-level data and health information collected at fine spatial resolutions can be used to identify heterogeneity that may be masked at higher levels of aggregation^4^. Ensuring that these epidemiologic data are accessible is vital to actively engage public health professionals and research communities in tailored research and response efforts. Additionally, tools and visualizations that engage and inform the general public on health outcomes and interventions (e.g., vaccines) in a digestible and transparent way are essential for successfully achieving a united and collaborative population-level response^5^.

In Canada, the first case of COVID-19 was publicly announced on January 25, 2020; and as of December 21, 2020, there have been a total of 519,292 reported cases and 14,373 reported deaths across 13 provinces and territories. More recently, the first vaccine doses were administered on December 14, 2020, and as of December 21, 2020 there have been 20,861 doses administered across the country. Much of the authority for healthcare delivery in Canada lies at the provincial and territorial level^6^, with each province/territory collecting data using their own bespoke reporting and surveillance information systems. However, this siloed collection limits standardized, comprehensive, and timely reporting of pan-Canadian health outcomes and interventions.

To address this critical data gap, the COVID-19 Canada Open Data Working Group (CCODWG) was created to systematically collect and report COVID-19 case and mortality data at the national, provincial/territorial, and sub-provincial level. Founded in March 2020, the CCODWG provides a comprehensive dataset of COVID-19 cases and deaths throughout Canada. Using standardized reporting fields, we provide fine scale geographic information for all data, and where possible, include demographics, any known transmission link, and travel history, among others. We also collect and report sub-national time series of COVID-19 recoveries, testing, and vaccine doses distributed and administered. The CCODWG compliments other COVID-19 tracking efforts that have tended to capture data only at national or the first administrative level (e.g., province), and can be combined with similar international efforts to assist in providing a clear picture of the global COVID-19 landscape at fine spatial resolutions^7–9^.

This real-time, open-access, sub-national epidemiological dataset aims to bring cohesion to the large volume of information generated from independent sources to enable robust pan-Canadian analyses. As the pandemic continues, the CCODWG datasets have the potential to be linked with new information to evaluate historical trends in disease transmission, conduct analyses in real-time, as well as forecast pandemic progression.

## Methods

The CCODWG collects information on Canadian COVID-19 confirmed, probable (e.g., non-lab confirmed, epidemiologically linked), and presumptive positive cases as well as deaths at the national, provincial/territorial, and health region level, with finer geographic resolution data (e.g., city, municipality) included for specific locales where available. Time series of COVID-19 recoveries, testing (including tests performed and persons tested), as well as vaccine doses distributed and administered are also recorded. Official definitions are used for cases, recoveries and tests; however, these vary across provinces/territories and have been modified over time. Information on the evolution of changes to important definitions across provinces and territories is available in a Technical Report^10^.

The CCODWG team is composed of over a dozen volunteer contributors with expertise in epidemiology, public health, and data science. Members were recruited through professional networks affiliated with the academic and research institutions of the management team. Upon joining, Data Curators received a standard onboarding process which included a welcome package that provided an overview of the project and objectives, a step-by-step instruction manual reviewing the project workflow, and an invitation to the online communication platform (www.slack.com). This was followed by an orientation meeting with a member of the Data Lead team, where a demonstration on the data entry process and workflow was provided. The group meets virtually during regular monthly meetings to maintain engagement and discuss issues and concerns.

All data are exclusively collected from publicly available sources, including government reports/releases and accredited news media. A hierarchy of preferred sources are used to identify case and mortality data, as well as corresponding demographic and exposure information. First, official government sources (such as press releases/press conferences from regional ministries of health) are reviewed in full and any COVID-19-related case and mortality announcements are identified as the gold standard for data inclusion. Second, additional information is identified using purposive search methods for COVID-19-related articles and online reports from accredited national and local news agencies. Finally, we identify updates reported by official social media accounts (e.g., Twitter) that are verifiably linked to governmental or public health institutions (e.g., ministries of health, chief medical officers of health), and these are included if no alternative sources are found as well as to supplement existing information. To improve data quality and auditability, the corresponding sources are required to be included as a reference for each data entry. Aggregated provincial/territorial recovery, testing, and vaccine dose distribution and administration data are also identified using this hierarchical process.

**Fig. 1:**
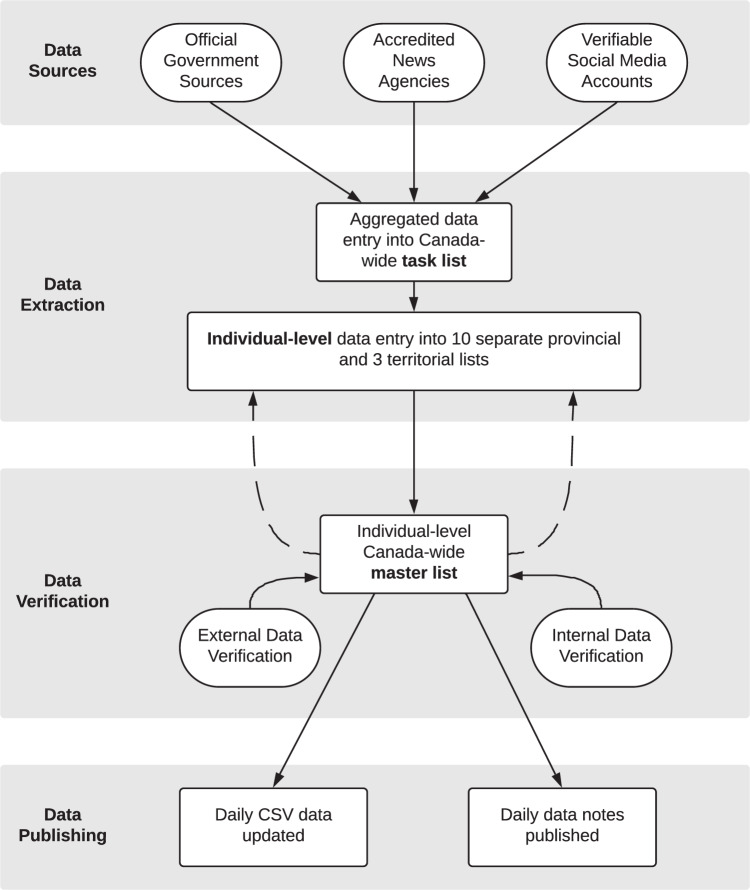
Overview of the COVID-19 Canada Open Data Working Group data collection, curation and verification process.

Data are manually identified and entered, which is necessary given the changing nature of information dissemination, non-machine-readable data sources, and changing web-links. Although many data sources have transitioned to machine-readable formats, jurisdictions continue to make changes to historical data that are difficult to incorporate into an automated process. Further, as jurisdictions do not use a singular date definition, the manual data entry process allows for a uniform ‘public report date’ across jurisdictions. To ensure consistency in data curation, we have established a streamlined data entry protocol using a stepwise data entry process that allows for these modifications to be incorporated, as required, while ensuring consistency in data curation over time and across jurisdictions (Fig. 1). Each day, Data Leads visit the aforementioned data sources to determine the total number of newly reported COVID-19 cases and deaths in each of Canada’s 10 provinces and three territories. Notably in Ontario, Canada’s most populous province, we refer directly to each of the 34 individual health region sources due to differences in data reporting between these and the Ontario Ministry of Health. The aggregated numbers for each region are added into a task list, from which members of the Data Curation team claim a task and enter the corresponding individual-level data using additional sources for demographics, where available. This stepwise process reduces risk of duplication and invalid entries. For each case and mortality, we collect data on the following, where available: the age and sex of the individual; the health region, province, and country; the report date and report week (using the date of the previous Sunday, to facilitate aggregation at the week-level); source of exposure such as travel or locally acquired; and source URL. Any additional epidemiologically relevant information (e.g., occupation, resident of a long-term care facility, staff or student at a school) is identified and compiled in a free text variable. Aggregated provincial/territorial recovery, testing, and vaccine dose distribution and administration data are entered as daily time series. All data entry processes are conducted using Google Sheets to enable collaboration and simultaneous editing and entry.

We have iteratively modified our data entry process in response to shifts in the level and type of data made available to the public over time. These changes are documented in a Technical Report^10^ and are also noted in the commit notes of our GitHub repository.

## Geo-Positioning

The finest spatial resolution consistently collected in our dataset is the health region level. In Canada, most provinces are subdivided into health regions along geographic or operationally meaningful boundaries^11^. The province of Prince Edward Island and the three territories are not subdivided, and each constitutes a single health region. Although these subdivisions are known by different names across provinces (e.g., ‘Zones’ in Nova Scotia, ‘Public Health Units’ in Ontario), we refer to them collectively as health regions for consistency. Throughout the course of the pandemic, health region boundaries have been modified in some jurisdictions, and we have updated our datasets and entry processes accordingly. These changes typically occur when a health region is subdivided into smaller units. In such cases, we aim to retroactively update case/mortality data to reflect the new health regions. If no, or only limited, historical health region data are publicly reported we aggregate the smaller units into the original larger units for consistency and include the smaller unit as additional information in a free text variable. Finer resolution spatial information (e.g., city) is also collected, where available. However, as this information is not readily available for the majority of cases/mortalities, it is not released in our public datasets. These data are available upon request. Population data for 2019 (total and age/sex stratified) corresponding to the health region boundaries used in the datasets are available from Statistics Canada and are linkable through the health region code (i.e., HR_UID)^12^.

## Data Records

A static version of the dataset up to December 21, 2020 is available on figshare^13^. The data are updated daily, and the latest version is available on our GitHub repository (https://github.com/ccodwg/Covid19Canada). Data are made available in a CSV format, facilitating importation and analysis in a variety of statistical software programs. Data are also available in JSON format from our API (https://opencovid.ca/api/). Given the pandemic is continuously evolving, all historical versions of the data and data notes are automatically archived on our GitHub repository. Daily data visualizations are made available through our interactive dashboard (https://art-bd.shinyapps.io/covid19canada/) and a further description of the project is available online at our project website (https://opencovid.ca/).

In the database, each row represents a single individual case. A description of the fields in the database is shown below and is also available as a codebook on the project GitHub repository. The fields capture information on demographics, geography, and source of infection.

**case_id:** National unique identifier. ID order does not necessarily reflect epidemiological progression, or reporting date, and should not be used to order cases in temporal progression.

**provincial_case_id:** Provincial unique identifier. ID order does not necessarily reflect epidemiological progression, or reporting date, and should not be used to order cases in temporal progression.

**age:** Age, in years. If a specific age is not given, then range provided (e.g., 50 s = 50–59). If no age is provided, Not Reported is specified. This variable may be updated retroactively as new information is released.

**sex:** Sex of the individual. If no sex is provided, Not Reported is specified. This variable may be updated retroactively as new information is released.

**health_region:** Name of the lowest health administrative division in which the case is reported. If no health region is provided or if a case is labelled as Out of Province, Not Reported is specified. This variable may be updated retroactively as new information is released.

**province:** Name of province/territory where case is reported. Cases reported to be Out of Province may be re-allocated between regions.

**country:** Name of country in which the case is reported (all Canada). By convention, travel-related cases are assigned to the country in which confirmation occurred - this is typically in the arrival country, rather than the site of infection. The locations of travel for such instances are described in ‘travel_history_country’.

**date_report:** Reported date (i.e., public announcement date) of case, recorded as DD-MM-YYYY. Although provinces/territories and health regions may have internal reporting dates (e.g., specimen collection, lab confirmation) these internal dates are not consistent across regions. Public reporting date is the only consistent date reported across each region and is used by default.

**report_week:** Week of report (beginning Sunday) of case, recorded as DD-MM-YYYY. As with ‘date_report’ this is based on public reporting date.

**travel_yn:** Binary flag to distinguish whether the case had a reported travel history associated with exposure. 0 denotes the case is not associated with travel exposure, 1 denotes the case is associated with travel exposure. If no information is provided, Not Reported is specified. This variable may be updated retroactively as new information is released.

**travel_history_country:** Open field listing country/countries of travel (or cross-provincial/territorial travel if within Canada) for those with travel exposure (i.e., 1 to ‘travel_yn’), If no information is provided, Not Reported is specified. This variable may be updated retroactively as new information is released.

**locally_acquired:** If no travel history (i.e., 0 to ‘travel_yn’), type of locally acquired infection (i.e., community, close contact) is recorded. This variable may be updated retroactively as new information is released.

**case_source:** URL identifying the source of case information.

**additional_info:** A free text variable for any additional information that may be informative about the case, such as the occupation of the patient (e.g., healthcare worker), whether they were identified through symptomatic or active surveillance, chronic conditions, the purpose of their travels, the hospital they were admitted to, etc.

**additional_source:** URL identifying the source of additional information provided in ‘additional_info’ variable.

**method_note:** Binary flag indicating whether retroactive updates or corrections to the dataset have been made. 0 denotes that no changes have been made to the case, 1 denotes that the case has been updated retroactively, 2 denotes a new ID has been assigned to case, and 3 denotes a modification based on the ‘additional_info’ variable.

At time of publication the dataset contains 519,292 case records and 14,373 mortality records from January 25, 2020 to December 21, 2020 across 92 health regions in all 10 Canadian provinces and 3 territories. Reference shapefiles with provincial and health region boundaries are prepared and maintained by ESRI Canada^14^. All shapefiles have a unique identifier for each health region component (HR_UID); correspondence between health region names used in our dataset and the HR_UID values are available via our GitHub repository. We do not use the shapefiles from Statistics Canada due to changes to the provincial health region boundaries over time.

## Technical Validation

The database is verified daily using a multi-step data validation process composed of complementary methodologies to identify and rectify any errors. First, a subset of the team that is not involved in entering the data manually reviews the daily numbers. This includes verifying that the case/mortality numbers from each province/territory/health region are correctly added to the task list. If these numbers align, the total number of cases/mortalities at the provincial level are verified against external government and public sources that report cumulative totals. The separate provincial/territorial Google Sheets are then aggregated into a Canada-wide master list for verification using cross tabulations of daily and cumulative totals, date formats, age and sex categories, as well as exposure sources.

Second, a validation script is run on this Canada-wide master list to compare the updated dataset to the previous day’s dataset. This process automatically flags any changes to historical data, which are highlighted for review and verification. These changes occur when additional information becomes available, cases are removed and/or reallocated to an alternate region, or other demographic or exposure sources are retroactively updated. This validation script also flags potential processing errors—including incompatible dates, age categories, spelling errors, etc. Discrepancies identified during the second data validation process are discussed collectively by Data Leads to reach a consensus issue resolution. The most commonly noted discrepancies include spelling errors, implausible/invalid age ranges or dates, which are updated in real-time. All automated data verification steps are conducted using R Statistical Software^15^. Code for these validation scripts is available on our GitHub repository.

Finally, notes pertaining to these daily data updates are included on the public dashboard website visualizing these data (https://art-bd.shinyapps.io/covid19canada/) as well as recorded in our daily GitHub commit message, which allows for a complete archive of data modifications. These may include notes to accompany updates to historical data, key contextual information (e.g., when a province redefines recovery, causing a sudden change in this value), and notices of changes to the data format (e.g., if a province redefines health region boundaries). We also provide CCODWG contact information for users to flag potential errors, which the management team consistently reviews, clarifies, and updates as needed. In addition to the daily verification process, the Data Lead team performs periodic internal audits to ensure sub-regional data validity against external sources to identify any error patterns in the data entry process that can be used to inform procedural changes among the Data Curation team.

## Usage Notes

**Fig. 2:**
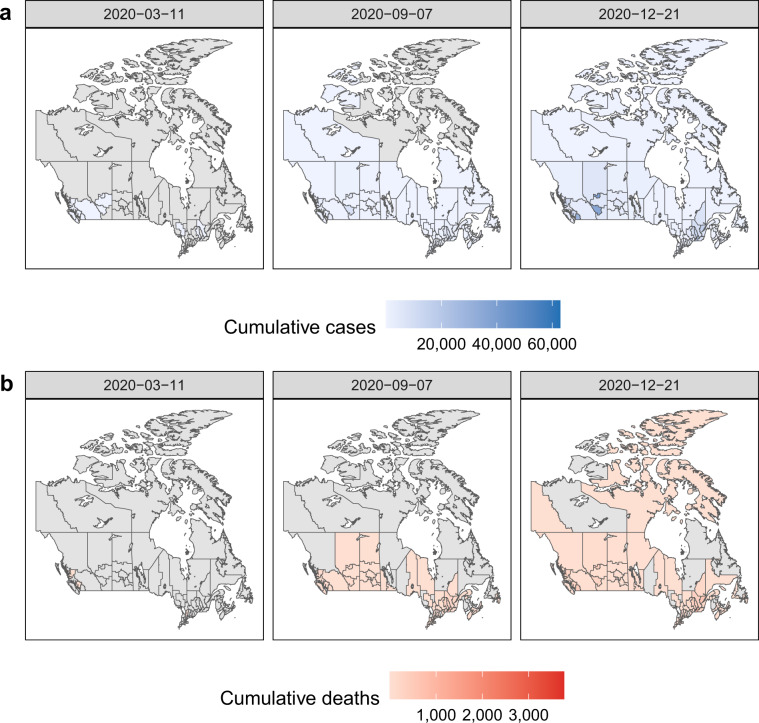
Health region-level map of cumulative reported COVID-19 cases and mortality in absolute counts. Cumulative (**a**) cases and (**b**) mortality in each health region of Canada from the first announced case on January 25, 2020 to WHO pandemic declaration [March 11, 2020]; to Labour Day weekend [September 7, 2020], to December 21, 2020. Values of 0 shown in grey.

**Fig. 3:**
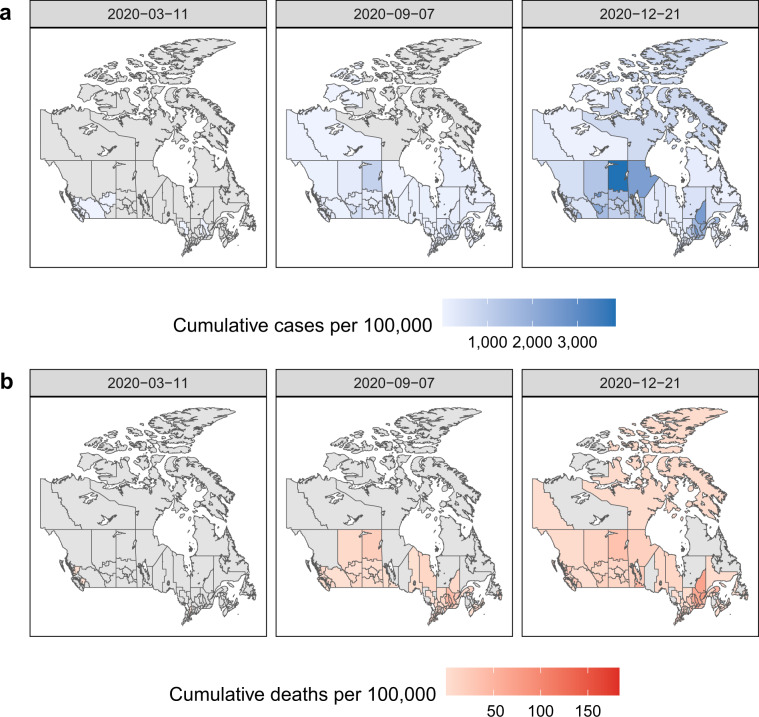
Health region-level map of cumulative reported COVID-19 cases per 100,000 and mortality per 100,000 over time. Cumulative (**a**) reported cases per 100,000 population and (**b**) mortality per 100,000 population in each health region of Canada from the first announced case on January 25, 2020 to WHO pandemic declaration [March 11, 2020], to Labour Day weekend [September 7, 2020], to December 21, 2020. Values of 0 are shown in grey. Health region populations correspond to 2019 estimates provided by Statistics Canada.

**Fig. 4:**
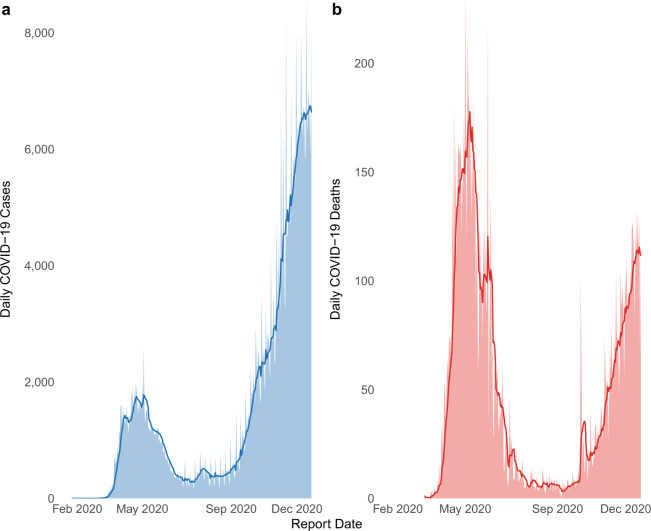
COVID-19 case and mortality epidemic curves in Canada. Daily reported (**a**) cases and (**b**) mortality with 7-day moving averages (bold line), across Canada since the first announced case on January 25, 2020 up to December 21, 2020.

The CCODWG dataset allows researchers, decision makers, journalists, and the general public to gain a better understanding of COVID-19 trends in Canada. The main strength of this dataset is the geographic scale at which case and mortality data are collected, and that these are provided in a near real-time manner. The concentration of data collection efforts at the health region level allows for the identification of COVID-19 hotspots at a fine spatial resolution, as well as changes in the locations of hotspots over time in absolute terms (Fig. 2) as well as per-capita (Fig. 3). Such analyses could be used to better understand geographical areas that may benefit from targeted control policies, such as lockdowns, and areas that may be able to modify existing policies. Additionally, we commenced this robust data collection effort at the start of the pandemic and to date, we have made daily public updates since March 15, 2020. By using a standardized data extraction and entry process with consistent report dates across regions and over time, we show how the COVID-19 pandemic has evolved across Canada (Fig. 4). This can be especially powerful when combined with information about key milestones or implementation of public health measures, including the rollout of vaccines that are also captured in this dataset. The data are not static; they are consistently being updated. The CCODWG updates the full dataset and the data dashboard daily, between 8 pm and 10 pm EST. Therefore, data should be downloaded as close as possible to the time of any analysis. Individual-level datasets are also available as aggregated daily time series at three spatial levels (national, provincial/territorial, and health region).

There are some limitations in solely using publicly available data. Firstly, very few official provincial/territorial government sources regularly provide demographic information for cases or mortalities in their daily press briefings, which leaves substantial gaps in the level of demographic information recorded. Generally, missing data on key socio-demographic and exposure variables increases with the duration of the pandemic and as cases/mortalities rise (Tables 1, 2). Health region information for cases and mortalities is the most complete variable within our dataset (>99%), although there is some variation across provinces and territories. Age, sex, city, and exposure source are missing in a large proportion of cases, however there are isolated provinces (e.g., PEI) that have relatively low levels of missingness that may be appropriate for local analysis. Among mortality data, information on age and sex is slightly more complete than case information, with isolated instances of zero missing data (e.g., Manitoba, New Brunswick, Newfoundland) depending on the time period considered. Furthermore, despite calls for harmonized and responsible collection of socio-demographic data^16,17^ (e.g., race, ethnicity, income), there have been very few jurisdictions to collect and report on this information to date. The collection of socio-demographic data in Canada has been limited in the absence of a national framework and in addition to concerns about data governance, accountability and protections from improper use of data. Public reporting of socio-demographic data, including race-based data is an important and necessary step in our ability to measure, monitor, and address health inequalities as a result of the COVID-19 pandemic^18^.

**Table 1:** Missing data for key demographic and exposure variables in the COVID-19 Canada Open Data Working Group case dataset.

Province/Territory	Age (%)	Sex (%)	Health Region (%)	City^1^ (%)	Exposure Source^2^ (%)
**January 25, 2020 to March 11, 2020**					
Alberta	0.00	0.00	0.00	5.26	0.00
British Columbia	2.17	0.00	13.04	93.48	0.00
Ontario	0.00	0.00	0.00	0.00	0.00
Quebec	75.00	50.00	0.00	25.00	0.00
Repatriated	100.00	100.00	100.00	100.00	0.00
**January 25, 2020 to September 7, 2020**					
Alberta	99.83	99.84	0.17	99.30	99.74
British Columbia	44.51	44.36	1.27	99.81	98.91
Manitoba	68.44	68.44	0.00	98.35	98.28
New Brunswick	9.38	90.62	0.00	82.29	46.88
NL	96.30	94.07	0.00	100.00	88.52
Nova Scotia	99.45	99.45	0.00	99.54	96.13
NWT	100.00	100.00	0.00	60.00	20.00
Ontario	95.85	96.16	0.00	97.79	96.95
PEI	23.53	3.92	0.00	84.31	1.96
Quebec	100.00	99.99	0.05	99.99	99.97
Repatriated	100.00	100.00	100.00	100.00	0.00
Saskatchewan	98.80	99.94	0.00	98.86	98.56
Yukon	100.00	100.00	0.00	80.00	40.00
**January 25, 2020 to December 21, 2020**					
Alberta	99.97	99.97	0.10	99.89	99.96
British Columbia	9.11	9.30	0.22	99.97	99.86
Manitoba	98.17	98.17	0.00	99.90	99.89
New Brunswick	4.33	96.89	0.00	94.12	62.11
NL	68.06	66.49	0.00	99.21	65.18
Nova Scotia	99.59	99.59	0.00	99.65	88.11
Nunavut	100.00	100.00	0.00	0.00	96.95
NWT	100.00	100.00	0.00	20.83	20.83
Ontario	97.27	97.41	0.00	97.96	98.40
PEI	16.48	3.30	0.00	87.91	7.69
Quebec	100.00	100.00	0.08	100.00	99.99
Repatriated	100.00	100.00	100.00	100.00	0.00
Saskatchewan	99.85	99.99	1.21	99.86	99.83
Yukon	100.00	100.00	0.00	64.41	44.07

**Note:** NL, Newfoundland and Labrador; NWT, North West Territories; PEI, Prince Edward Island. ^1^City-level spatial information is collected, where available. However, as this information is not readily available for the majority of cases/mortalities, it is not released in our public datasets. These data are available upon request.^2^Exposure source data is captured within the data field travel_yn.

**Table 2:** Missing data for key demographic and exposure variables in the COVID-19 Canada Open Data Working Group mortality dataset.

Province/Territory	Age (%)	Sex (%)	Health Region (%)
**January 25, 2020 to March 11, 2020**			
British Columbia	0.00	0.00	0.00
Ontario	0.00	0.00	0.00
**January 25, 2020 to September 7, 2020**			
Alberta	61.57	61.57	0.00
British Columbia	97.16	96.21	0.00
Manitoba	0.00	0.00	0.00
New Brunswick	0.00	50.00	0.00
NL	0.00	0.00	0.00
Nova Scotia	81.54	81.54	0.00
Ontario	92.95	93.16	0.00
Quebec	98.67	99.69	0.00
Saskatchewan	0.00	66.67	0.00
**January 25, 2020 to December 21, 2020**			
Alberta	62.67	62.67	0.00
British Columbia	99.08	98.95	0.00
Manitoba	0.00	0.00	0.00
New Brunswick	0.00	87.50	0.00
NL	0.00	0.00	0.00
Nova Scotia	81.54	81.54	0.00
Nunavut	100.00	0.00	0.00
Ontario	94.56	94.70	0.00
Quebec	99.01	99.77	0.00
Saskatchewan	11.48	93.44	0.00
Yukon	100.00	100.00	0.00

**Note:** NL, Newfoundland and Labrador.

Recently some provincial ministries of health, including those of Ontario, British Columbia, and Alberta, have released public line lists of cases. The CCODWG is actively developing probabilistic linkage algorithms to match these cases to our datasets to improve data completeness. However, discrepancies between the date cases are publicly reported (i.e., report date) and the date that provincial health authorities became aware of each case (often the date of positive test or onset of symptoms) can result in merging issues between these datasets. Secondly, health authorities frequently make changes to previously announced cases without providing specific information about exactly which case(s) on which day(s) have been altered. Therefore, we are often required to make assumptions about which cases to change. When making such edits we prioritise matching cumulative health region totals, which can lead to distortions in daily case and mortality counts; we recommend using 7-day rolling averages to evaluate daily trends. To ensure transparency in all instances of retroactive data modification, we record updates as additional information in a free text variable and provide compiled daily public data notes and explanations to users. Additionally, work by the CCODWG is ongoing to enable data users to substitute officially released provincial/territorial datasets, where available, into our dataset with the understanding that the date definitions across jurisdiction datasets may not be consistent. With respect to the vaccine administration data, we further note that doses administered does not equate to the number of people vaccinated as currently approved vaccines require two doses delivered at least several weeks apart^19^.

As the pandemic continues, changes in the public reporting of cases, mortalities, recoveries, testing, vaccine doses distributed, or vaccine doses administered may continue to occur. While individual-level data collection will end on May 31, 2021, time series data collection is planned to continue as long as the data are being reported. The CCODWG continues to ensure that any such modifications are accurately reflected in the datasets and additional context is provided to users. As the COVID-19 pandemic in Canada progresses, we intend to continue updating, curating, and releasing these datasets.

## Data Availability

Data are available in CSV format from our GitHub repository: https://github.com/ccodwg/Covid19Canada. Data are also available in JSON format from our API: https://opencovid.ca/api/. All code used to generate and verify the data are also available from our GitHub repository: https://github.com/ccodwg/Covid19Canada/tree/master/scripts. All data and code required to reproduce the figures and tables in this manuscript are available in the following GitHub Repository: https://github.com/ccodwg/ccodwg-scientific-data. All code can be run using any recent version of R.
